# Self-assembling multilayer MSC-sheet promotes wound healing increasing M2 macrophage polarization

**DOI:** 10.1038/s41598-025-33482-w

**Published:** 2025-12-26

**Authors:** Qiannan Zhao, Michiharu Sakamoto, Jinya Liu, Yuanjiaozi Li, Hitoshi Kusano, Eiichi Sawaragi, Hang Dong, Hiroki Yamanaka, Naoki Morimoto

**Affiliations:** 1 Department of Plastic and Reconstructive Surgery, Faculty of Medicine, Graduate School of Medicine, Kyoto University, 54 Shogoin, Kawahara-cho, Sakyou-ku, Kyoto, 606-8507 Japan; 2 PharmaBio Corporation, Kawasaki City, Kanagawa Japan

**Keywords:** Human mesenchymal stem cells, Multilayer MSC-sheet, MSC-suspension, Wound healing, Macrophage infiltration, Angiogenesis, ECM, Biotechnology, Cell biology, Diseases, Stem cells

## Abstract

Mesenchymal stem cell (MSC) therapy holds promise for treating chronic wounds. However, low cell density in the target tissue and short retention time limits its efficacy. This study investigated the therapeutic effects of the self-assembling multilayer MSC-sheet in a murine wound model, focusing on wound healing and macrophage infiltration. The MSC-sheet was prepared from human subcutaneous adipose tissue and characterized by histology and RNA sequencing. In vivo, the α-MEM (control), MSC-suspension, and MSC-sheet were applied to full-thickness skin defects in C57BL/6 J Jcl mice. On days 7, 14, and 21, the remaining wound area, neoepithelialization, granulation tissue, cell retention time, angiogenesis, and macrophage infiltration were evaluated using hematoxylin and eosin, Azan, anti-human nucleoli (HN), anti-CD31, anti-CD68, and anti-CD163 staining. Macrophage polarization was evaluated by RT-PCR on days 3, 7, and 14. The MSC-sheet formed a multilayer structure (6–7 layers) containing extracellular matrix (ECM). RNA sequencing identified 756 differentially expressed genes compared with MSC-suspension, highlighting pathways related to wound healing, anti-inflammation, angiogenesis, and macrophage polarization. In vivo, both the MSC-sheet and MSC-suspension significantly reduced the remaining wound area (days 7 and 14), increased neoepithelialization (day 14), granulation tissue formation (day 7), angiogenesis (day 7) and macrophage infiltration (days 7 and 14) compared to the control group. The MSC-sheet maintained superior MSC retention and greater M2 macrophage induction than suspension, confirmed by RT-PCR. The MSC-sheet and MSC-suspension accelerated wound healing and promoted M2 macrophage polarization. The MSC-sheet, with its multilayer structure and ECM, outperformed suspension in sustaining MSCs and enhancing M2 infiltration. These findings position the self-assembling multilayer MSC-sheet as a promising, scaffold-free cell delivery platform for chronic wounds, with potential scalability and clinical applicability for future translational use.

**Supplementary Information:**

The online version contains supplementary material available at 10.1038/s41598-025-33482-w.

## Introduction

Wound healing is a complex biological process orchestrated by intrinsic mechanisms within the skin. Surgical incisions and minor lacerations can result in chronic wound formation due to uncontrollable inflammatory reactions in the wound region or underlying diseases^1^. The global incidence of chronic wounds is rising annually at a rate of 6–7%^2^, posing substantial challenges to patients’ quality of life, clinicians, and socioeconomics^3^.

Mesenchymal stem cell (MSC) therapy is promising for chronic wound healing because of its inherent characteristics, such as proliferation, multiple differentiations, secretion of various growth factors, homing into target tissues, subsequent promotion of angiogenesis, regulation of macrophage infiltration, and remodeling of the extracellular matrix (ECM)^1,4,5^. Notably, two aspects of stem cell therapy should be addressed: delivering a sufficient number of stem cells to the target site and ensuring their sustained presence and activity to facilitate tissue regeneration^6–8^. Conventional MSC therapy typically involves the local or intravenous injection of cell suspensions harvested from culture dishes through enzymatic digestion. While this method is commonly employed, it has drawbacks; in particular, cells dissociated through enzymatic digestion require a significant amount of time to recover their cellular activity, often resulting in the loss of ECM, cell-to-cell connections, and stem cell-homing capabilities^7,9,10^. In addition, the intravenous injection method is relatively inefficient, as only 7.4 × 10^2^ MSCs were detected in the skin wounds of mice that received 1 × 10^6^ MSCs intravenously^11^. Although the development of cell sheets that integrate MSCs with biodegradable materials has considerable potential, it faces several challenges, including material strength, porous structure, and manufacturing process. These issues can potentially hinder capillary formation, exacerbate microbiological contamination, and trigger chronic inflammatory responses owing to material degradation^12–14^.

Cell sheet technology has emerged as a unique therapeutic approach for overcoming these issues. This technique involves the aggregation of cells along with their ECM in a sheet-like form, facilitating transplantation and exhibiting strong adhesive properties^15^. MSC-sheet technology offers several advantages, including the retention of endogenous cell junctions and ECM^16,17^, preservation of cell-adhesive proteins for stable biological adhesion without sutures^18^, and maintenance of natural cellular environments that promote cell viability^19^. The MSC-sheet has demonstrated efficacy in various medical applications, including heart tissue repair^20^, ocular surface reconstruction^21^, prevention of esophageal stricture^22^, colon anastomotic leakage^18^ and promotion of lymphangiogenesis^23^.

Recent studies have demonstrated the therapeutic benefits of the MSC-sheet for wound healing^13,24,25^. However, previously reported MSC-sheets often have a 1–3-cell layer structure^13,17,25^, making it difficult to achieve optimal cell quantity in wounds. Furthermore, the mechanisms underlying the therapeutic effects of the MSC-sheet across various phases of wound healing have not been fully elucidated. Therefore, in this study, we utilized an innovative cell-sheeting method that involves seeding cells at a high density in culture dishes to create a self-assembling MSC sheet without relying on any scaffold material and investigated their therapeutic effects in a mouse wound model. Unlike conventional suspension injection or scaffold-based grafts, this scaffold-free, multilayer MSC-sheet preserves endogenous ECM and cell–cell interactions, thereby improving cell retention, viability, and immunomodulation. Importantly, its reproducible preparation process highlights the potential for clinical scalability. Our findings therefore not only provide mechanistic insights into wound healing but also underscore the translational feasibility of MSC-sheet as a novel therapeutic platform for chronic wound management.

## Materials and methods

### Cell culture

Human adipose tissue-derived MSCs were purchased from Essent Biologics (Colorado, USA). After expansion to passage 2 (P2), the cells were mixed with a solution of CP-1 cryoprotectant (Kidoh Industries Co., Ltd., Tokyo, Japan) and albumin (Sigma-Aldrich, St. Louis, USA), and subsequently cryopreserved at temperatures below − 150 °C. The surface antigen profiles of the cells were characterized and confirmed to meet MSC criteria^26^.

### Preparation of the MSC-sheet and MSC-suspension

After thawing, cryopreserved human allogeneic adipose-derived MSCs were washed with phosphate-buffered saline (PBS) and cultured for one additional passage before preparing the cell sheet. The MSC-sheet was produced and supplied by PharmaBio Corporation using an advanced, newly developed cell incubation technique^26,27^. The specific manufacturing protocols, including culture duration, initial seeding density, and medium composition, are confidential intellectual property of PharmaBio Corporation and cannot be disclosed. During cell-sheet preparation, an equivalent number of MSCs required for a single cell sheet (approximately 5.5 × 10^5^ cells) were isolated from the remaining MSCs and used to prepare the MSC-suspension. Both the MSC-sheet and MSC-suspension were stored in α-MEM medium supplemented with albumin, which served as a preservation medium, and transported at 2–8 °C.

### RNA sequence

Total RNA was extracted using the RNeasy MittElute Cleanup Kit (74,204), according to the manufacturer’s instructions. RNA quality was initially assessed using a NanoDrop ND-1000 to measure RNA concentration, and then further verified by agarose gel electrophoresis on a bioanalyzer. Difference analysis was performed between the MSC-suspension and MSC-sheet to identify differentially expressed genes (DEGs). To ensure reliability, DEGs with |logFC|> 2 and *P* value < 0.05 were considered significant. R software (version 4.4.1) package “ggplot2” was used for the volcano plot and heatmap. To investigate the potential interaction networks of the target proteins, the “Search Tool for the Retrieval of Interacting Genes” (STRING) was used to map the DEGs. Notably, interactions with a combined score over 0.9 were considered high confidence. Cytoscape” (version 3.10.3) was used to identify the hub nodes for DEGs. “MCODE” algorithm was used to cluster high-density regions in the protein–protein interaction (PPI) network. To further elucidate the pathways enriched in the DEGs, functional annotation and enrichment analyses were performed. “AnnotationDbi,” “org.Hs.eg.db” and “clusterProfiler” packages in R software were used to perform GO and KEGG pathway enrichment. Besides, “ggplot2″ was used to visualization (*p*-values < 0.05).

### Preparation of animal experiments

All experimental protocols involving animals were approved by the Animal Research Committee of the Kyoto University Graduate School of Medicine (permit no. Med Kyo 23109). All procedures were performed in accordance with relevant institutional guidelines and regulations (Guidelines for Animal Experimentation of Kyoto University, Japan), and every effort was made to minimize animal suffering and reduce the number of animals used. This study is reported in accordance with the ARRIVE guidelines (https://arriveguidelines.org).

A total of 108 C57BL/6 J Jcl mice (male, 8–9 weeks old; purchsed from CLEA Japan, Inc., Tokyo, Japan) were housed one per cage and maintained in a temperature-controlled animal facility. These mice were randomly allocated to three groups—namely, the control, MSC-suspension, and MSC-sheet groups. At 1 day prior to surgery, the hair on the back of each mouse was shaved using an electric shaver (Thrive; Daito Electric Machine Ind., Co., Ltd., Osaka, Japan) and depilated using a depilation cream (Kracie, Tokyo, Japan). All painful procedures were performed under general anesthesia using isoflurane (Pfizer Inc., Kyoto, Japan) in spontaneously breathing animals, maintaining a concentration of 1.5–2% for an appropriate depth of anesthesia.

As to the precise surgical procedure, a donut-shaped silicone skin splint (outer diameter: 18 mm; inner diameter: 12 mm; thickness: 0.5 mm; Fuji System Corp., Tokyo, Japan) was initially attached to the skin with binding adhesive (Aron Alpha; Daiichi Sankyo, Osaka, Japan) and secured with 5–0 nylon sutures (Bear Corporation, Osaka, Japan) to prevent wound contraction. Subsequently, an 8 mm diameter full-thickness skin defect was created at the center of the applied skin splint using an 8 mm biopsy punch (Kai Industries Co., Ltd., Tokyo, Japan) and scissors.

In the MSC-sheet group, a piece of the MSC-sheet was placed at the center of the wound along with 50 μl of preservation medium. In the MSC-suspension group, 50 μl of the MSC-suspension was applied onto the wound and allowed to settle for 30 min. In the control group, 50 μl of α-MEM medium without MSC was applied. The wounds were covered with a silicone mesh sheet (2 × 2 cm; SI mesh, ALCARE Co., Ltd., Tokyo, Japan), sutured to the marginal skin with 5–0 nylon, covered with gauze, and secured with a surgical tape bandage (Silkytex, ALCARE Co., Ltd., Tokyo, Japan) to prevent contamination and mechanical stress (Fig. 3A). Following these procedures, the mice were individually housed in cages in an institutional animal facility. Histological examinations were performed on days 7, 14, and 21 (n = 6 per time point), and reverse transcription polymerase chain reaction (RT-PCR) analyses were conducted on days 3, 7, and 14 (n = 6 per time point). The sample size for all experiments was predetermined based on our prior experience. No samples were excluded from this study. All animals were allocated using simple randomization. Data analysis was conducted in a single-blind manner, where researchers were unaware of group allocation during both the experiment and outcome assessment. This work has been reported in accordance with the ARRIVE guidelines 2.0.

### Tissue harvesting and histological preparation

Wound evaluation and tissue collection were performed at 7, 14, and 21 days after surgery. At each time point, six mice per group were euthanized by carbon dioxide gas inhalation. The wounds images were captured using a digital camera (Sony Corporation, Tokyo, Japan). Wound specimens, including surrounding tissues, were harvested and fixed in 10% buffered formalin (FUJIFILM Wako Pure Chemical Co., Ltd., Osaka, Japan), embedded in paraffin, and sectioned into 5 μm slices along the central axis of the wound. Following deparaffinization and rehydration, the sections were stained with hematoxylin and eosin (H&E) to assess the neoepithelial length and with Azan staining to evaluate granulation tissue formation.

For immunofluorescence (IF) staining to evaluate MSC retention, sections were first subjected to deparaffinization, rehydrated, and subjected to heat-induced antigen retrieval in 0.001 M ethylenediaminetetraacetic acid (EDTA, pH 8.0, Nacalai Tesque, Inc., Kyoto, Japan) at 90 °C for 15 min. Sections were then blocked with 5% Goat Normal Serum (CAT#ab7481, Abcam, Cambridge, UK) for 1 h and incubated overnight at 4 °C with anti-Human Nucleoli (HN) primary antibody (1:200, CAT#ab190710, Abcam, Cambridge, UK). Afterward, sections were treated with fluorescent secondary antibodies (Alexa Fluor 594, CAT#A-11032, Invitrogen, Thermo Fisher Scientific, Waltham, MA, USA) and 4′,6-diamidino-2-phenylindole (DAPI) (CAT#62248, Thermo Fisher Scientific, Waltham, MA, USA).

For immunohistochemical (IHC) staining to elucidate the capillaries and macrophages, sections were subjected to antigen retrieval, followed by overnight incubation at 4 °C with primary antibodies, including anti-CD31 (1:10,000, CAT#ab182981, Abcam, Cambridge, UK), anti-CD68 (1:5000, CAT#ab125212, Abcam, Cambridge, UK), and anti-CD163 (1:4000, CAT#ab182422, Abcam, Cambridge, UK). A polymeric reagent (Simple Stain Mouse MAX PO; Nichirei Biosciences Inc., Tokyo, Japan) was used as the secondary antibody. The sections were subsequently exposed to 3,3′-diaminobenzidine (DAB) (Nichirei Biosciences Inc., Tokyo, Japan) and counterstained with hematoxylin. Histological photomicrographs were captured and analyzed using the BZ-X800 Analyzer software (Keyence Corp., Osaka, Japan).

### Assessment of the remaining wound area

On days 7, 14, and 21, wound edges were traced from photographs to assess non-epithelialized areas using ImageJ software (version 1.53, National Institutes of Health, Bethesda, Maryland, USA).

### Assessment of epithelialization and granulation tissue formation

The neoepithelial length and granulation tissue area were measured on days 7, 14, and 21. Neoepithelial length was determined by measuring the distance from the nearest hair follicle to the edge of the epithelium on both sides of the wound in H&E-stained sections. Measurements from each side were subsequently combined to represent the total neoepithelial length.

The granulation tissue area located above the muscle layer was quantified from the Azan-stained sections. In these sections, the immature fibrous connective tissue within the granulation tissue appears light blue, providing a clear distinction from the dark blue staining of the surrounding intact dermis.

### Assessment of the persistence of transplanted MSC

To evaluate the persistence of MSC transplanted into the wound tissue, an immunofluorescent staining technique targeting HN was employed on the biopsied samples collected on day 7. The presence of red fluorescence localized within the nucleus (identified by DAPI staining) indicated HN positivity, confirming the presence of transplanted MSC.

### Assessment of newly formed capillary number and area

The number and area of newly formed capillaries in the granulation tissue were assessed on days 7, 14, and 21 using sections stained with the vascular marker, anti-CD31^28^. Consistent with previous studies^29^, a threshold was set for the brown color tone of DAB staining. Capillaries were identified as spots with a color density surpassing this threshold, and both the number and area of the capillaries were quantified using a BZ-X800 Analyzer (Keyence Corp., Osaka, Japan).

### Assessment of macrophage infiltration

Pan-macrophage and M2 macrophage numbers were assessed on days 7, 14, and 21 using sections stained with the pan-macrophage marker anti-CD68 and anti-inflammatory M2 macrophage marker anti-CD163, respectively^30^. Quantification was conducted using a BZ-X800 Analyzer (Keyence Corp., Osaka, Japan), following the same procedure used for capillary analysis. The M2 ratio was calculated as the ratio of CD163-positive cells to CD68-positive cells.

### Comparison of RNA expression in wound tissue by RT-PCR

Wound tissue was collected and total RNA was extracted using the RNeasy Plus Universal Mini Kit (CAT#73,404, QIAGEN). RNA purity and concentration were evaluated using a NanoDrop 2000 spectrophotometer (Thermo Fisher Scientific). The purified RNA was reverse-transcribed into cDNA using the SuperScript™ VILO™ cDNA Synthesis Kit (CAT#11754050, Invitrogen™) in a thermal cycler, LifeECO (NIPPON Genetics Co., Ltd., Tokyo, Japan).

The synthesized cDNA was diluted to 500 ng/μL with RNAse-free water and used as a template. TaqMan® Gene Expression Assays were employed on Quantstudio3 Real-time PCR System (Applied Biosystems, USA). Each reaction mixture (20 μL) contained 2 μL of cDNA template, 10 μL of TaqMan® FAST Advanced Master Mix (CAT#4444557, Applied Biosystems™), 1 μL of Taqman Gene Expression Assay, and 7 μL of RNAse-free water in MicroAmp™ Optical 96-Well Reaction Plates (Applied Biosystems™, Foster, CA, USA). The thermal cycling conditions were as follows: 95 °C for 10 min, followed by 50 cycles of 95 °C for 20 s and 60 °C for 1 min. glyceraldehyde-3-phosphate dehydrogenase (GAPDH) served as an internal control for normalizing target gene expression. TaqMan Gene Expression Assays were performed for inducible nitric oxide synthase (INOS) (Assay ID: Mm00475988_m1), arginase-1 (ARG1) (Assay ID: Mm00440502_m1), Vascular Endothelial Growth Factor A (VEGFA) (Assay ID: Mm00437306_m1), and glyceraldehyde-3-phosphate dehydrogenase (GAPDH) (assay ID: Mm99999915_g1). The relative fold expression was calculated using the comparative Ct method by 2-ΔΔCt.

### Statistical analysis

All data are presented as mean ± standard deviation. One-way ANOVA followed by LSD post-hoc analysis was conducted for comparisons among multiple groups. Statistical analyses were performed using IBM SPSS Statistics version 28, and visualization was performed using GraphPad Prism version 10. Statistical significance was determined using a probability (P) P-value of < 0.05.

## Results

### Evaluation of MSC-sheet properties

**Fig. 1:**
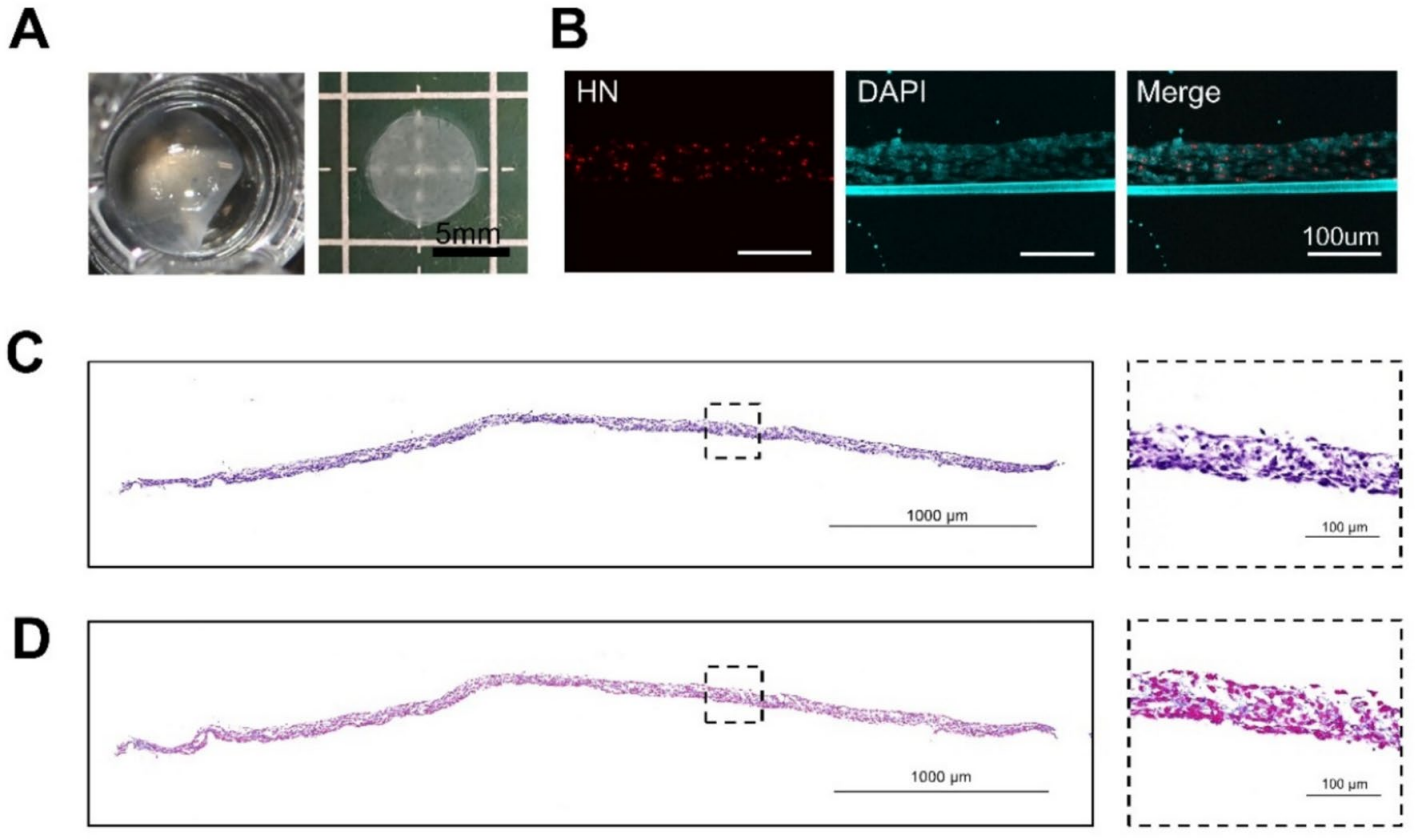
Characteristics of the MSC-sheet. (**A**) Gross observation of the MSC-sheet, showing a diameter of 6 mm. (**B**) Immunofluorescence staining with an anti-human nucleolus (HN) antibody (red) and DAPI (cyan), confirming the human origin of the cells. Scale bar: 100 µm. (**C**) H&E staining showing 6–7 layers of cells on the MSC-sheet. Scale bars: 1000 µm (overview) and 100 µm (inset). (**D**) Azan staining demonstrating the presence of extracellular matrix (ECM) within the MSC-sheet. Scale bars: 1000 µm (overview) and 100 µm (inset).

The characteristics of the MSC-sheet were assessed by visual observation, histological analysis, and immunofluorescence (Fig. 1). Visual observation revealed that the MSC-sheet was 6 mm in diameter and had a translucent membrane structure of uniform thickness that could be easily grasped using surgical forceps (Fig. 1A). Immunofluorescence staining using an anti-human nucleolar (HN) antibody (red) and DAPI (cyan) confirmed the human origin of the cells (Fig. 1B). H&E staining revealed that the MSC-sheet consisted of 6–7 cell layers (Fig. 1C). In this study, we define “multilayer” based on this histological observation of piled-up cell strata supported by their own ECM, rather than a specific multi-step stacking manufacturing process. Additionally, Azan staining demonstrated the presence of an abundant ECM throughout the MSC-sheet (Fig. 1D).

### Identification of DEGs between the MSC-sheet and MSC-suspension

**Fig. 2:**
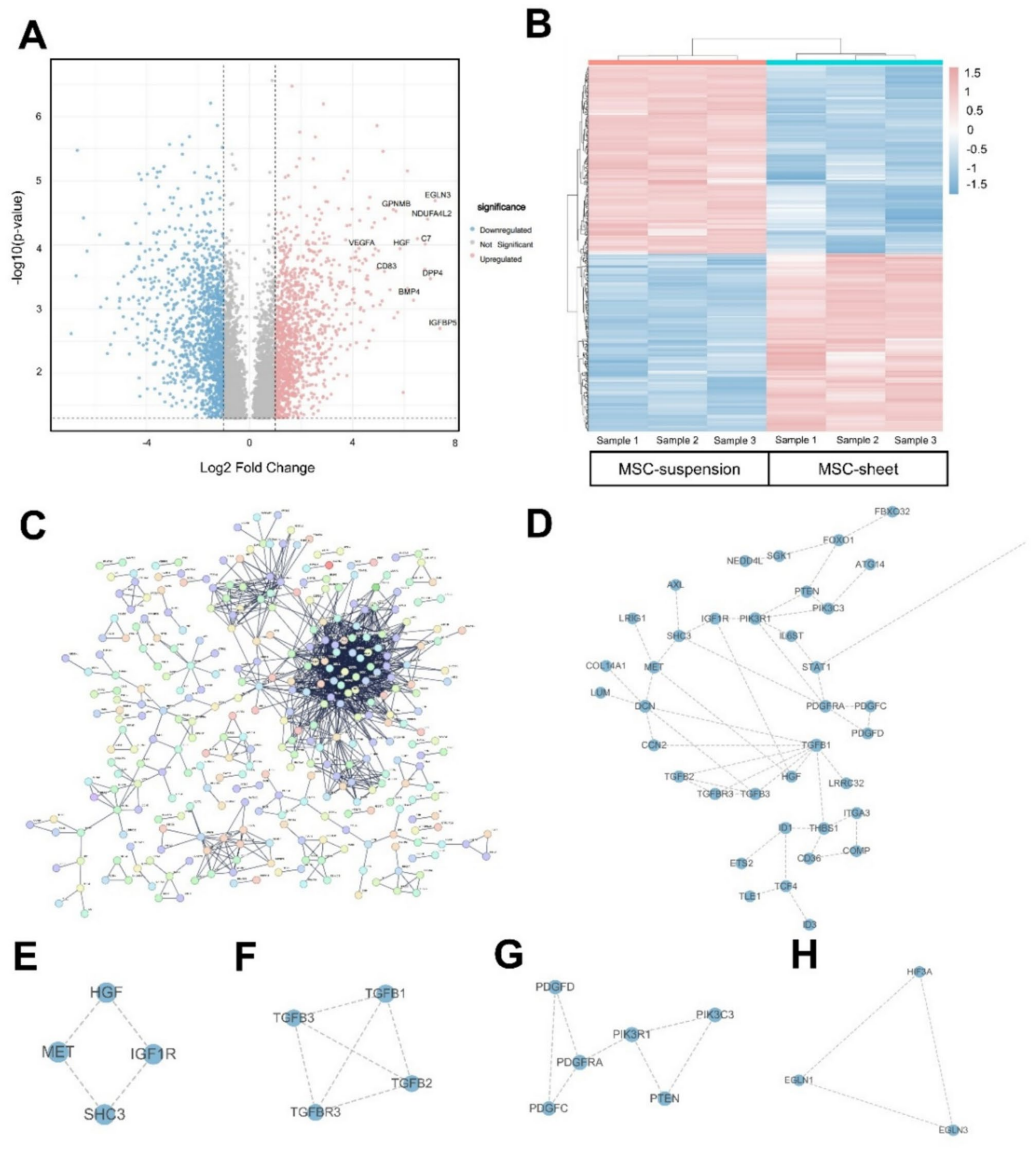
RNA-seq analysis and functional enrichment of differentially expressed genes (DEGs) between the MSC-sheet and MSC-suspension. (**A**) Volcano plot displaying the DEGs identified between the MSC-sheet and MSC-suspension. Red dots represent upregulated genes, blue dots represent downregulated genes, where gray dots represent non-significant genes. Key genes associated with angiogenesis (VEGFA, HGF, and GPNMB), anti-inflammatory responses (BMP4 and CD83), and cellular regulation (EGLN3, NDUFA4L2, DPP4, IGFBP5, and C7) were identified. (**B**) Heatmap visualizing 756 DEGs, including 364 upregulated and 392 downregulated genes, in the MSC-sheet group. Red represents upregulation and blue represents downregulation. (**C**) Protein–protein interaction (PPI) network constructed using STRING and visualized in Cytoscape, illustrating the global connectivity of the DEGs. Nodes represent genes, and edges represent interactions. Dense clusters suggested functionally related modules. (**D**–**H**) Zoomed-in views of specific gene clusters identified in the PPI network using the MCODE tool in Cytoscape.

RNA-seq analysis revealed 21,445 genes in the MSC-sheet and MSC-suspension samples. Subsequent differential expression analysis revealed 756 DEGs, including 364 upregulated and 392 downregulated genes, in the MSC-sheet group compared with those in the MSC-suspension group. A volcano plot (Fig. 2A) was used to illustrate the distribution of DEGs with key genes related to angiogenesis (VEGFA, HGF, and GPNMB), anti-inflammatory responses (BMP4 and CD83), and cellular regulation (EGLN3, NDUFA4L2, DPP4, IGFBP5, and C7). A heatmap (Fig. 2B) further visualized the 756 DEGs, in which upregulated and downregulated genes are shown in red and blue, respectively.

### Wound healing-related PPI network

The 756 DEGs identified between the MSC-sheet and MSC-suspension were imported into Cytoscape to construct a PPI network (Fig. 2C) retrieved from the STRING database with a network confidence coefficient of 0.9. To further uncover gene clusters potentially involved in wound healing, the MCODE tool was used to identify modules within the network. In total, 20 gene clusters were obtained, four of which (clusters 7, 14, 19, and 20) showed strong associations with wound healing (Fig. 2D–H). Specifically, cluster 7 was enriched in the TGF-β signaling pathway, cluster 14 in the HIF-1 signaling pathway, and clusters 19 and 20 in pathways related to M2 macrophage polarization and anti-inflammatory responses. These findings indicate that the MSC-sheet configuration may potentiate wound healing via multiple mechanisms, including immunomodulation and enhanced tissue repair processes.

### Enrichment analysis

**Fig. 3:**
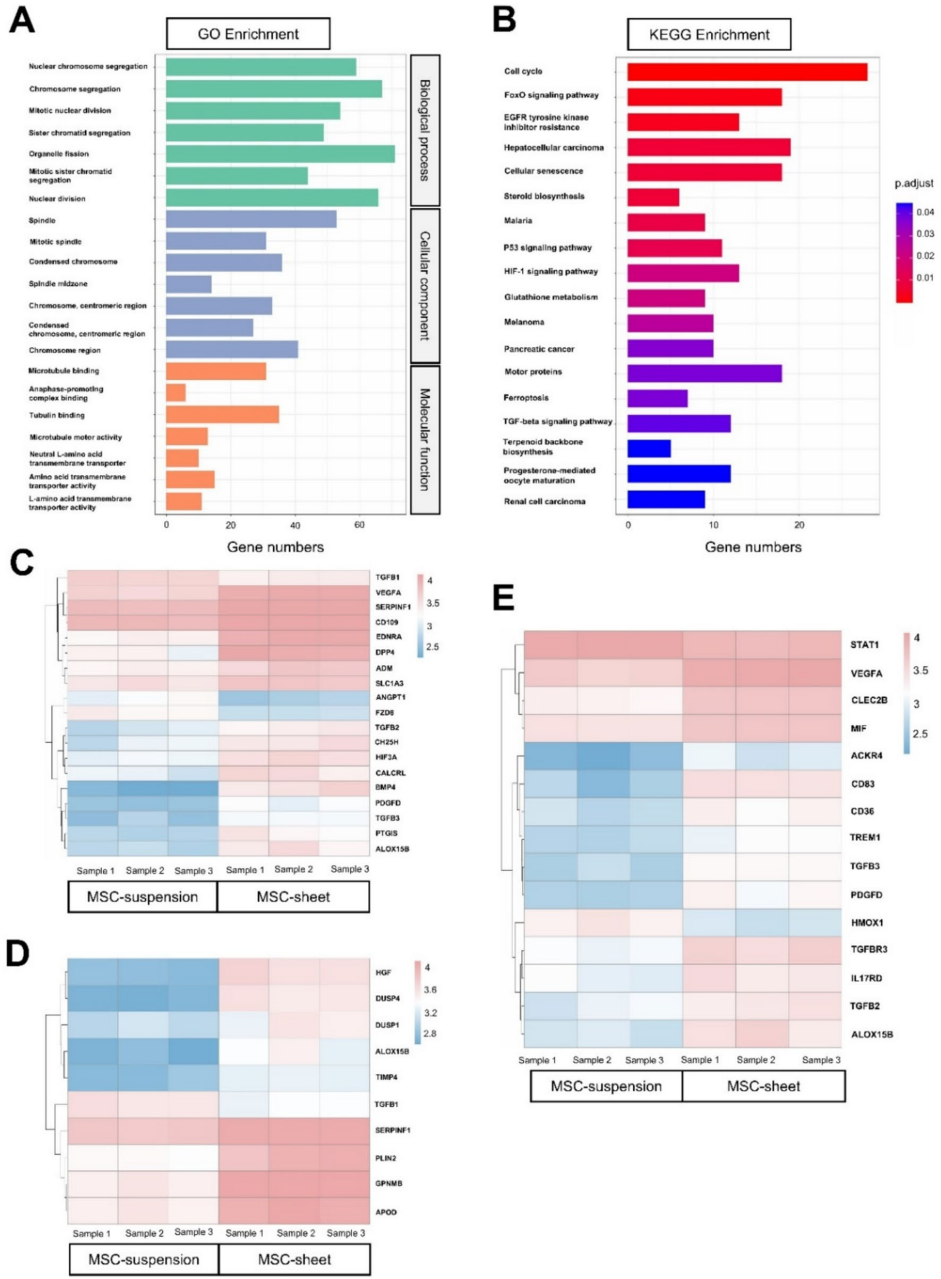
GO and KEGG enrichment analysis of differentially expressed genes (DEGs) and heatmap representation of key genes between the MSC-sheet and MSC-suspension. (**A**) GO enrichment analysis of DEGs categorized into three domains: biological processes, cellular components, and molecular functions. Nuclear chromosome segregation, organelle fission, spindle assembly, tubulin binding, and transmembrane transporter activity were significantly enriched. (**B**) KEGG pathway enrichment analysis highlighting key pathways such as cell cycle, FoxO signaling, TGF-β signaling, and HIF-1 signaling. Pathway image generated using KEGG (Kanehisa Laboratories). Citation: Kanehisa M, et al. KEGG: Kyoto Encyclopedia of Genes and Genomes (see https://www.kegg.jp/kegg/kegg1.html). (**C**) Heatmap depicting expression levels of genes associated with angiogenesis. (**D**) Heatmap illustrating genes related to anti-inflammatory responses. (**E**) Heatmap focusing on genes involved in M2 macrophage polarization.

To further delineate the biological relevance of DEGs, GO and KEGG enrichment analyses were performed (Fig. 3A, B). The GO enrichment results revealed the significant involvement of DEGs in nuclear chromosome segregation, organelle fission, spindle assembly, tubulin binding, and transmembrane transporter activity (Fig. 3A). Correspondingly, KEGG pathway analysis underscored key signaling pathways such as the cell cycle, FoxO, TGF-β, and HIF-1 (Fig. 3B). Genes associated with angiogenesis, anti-inflammatory responses, and M2 macrophage polarization were selected for heatmap visualization (Fig. 3C–E). Compared with the MSC-suspension group, the MSC-sheet group exhibited enhanced pro-angiogenic capacity (Fig. 3C), stronger anti-inflammatory effects (Fig. 3D), and more pronounced ability to promote M2 macrophage polarization (Fig. 3E), thereby suggesting a broader immunomodulatory and tissue-reparative potential afforded by the MSC-sheet configuration.

### Macroscopic evaluation of wound healing

**Fig. 4:**
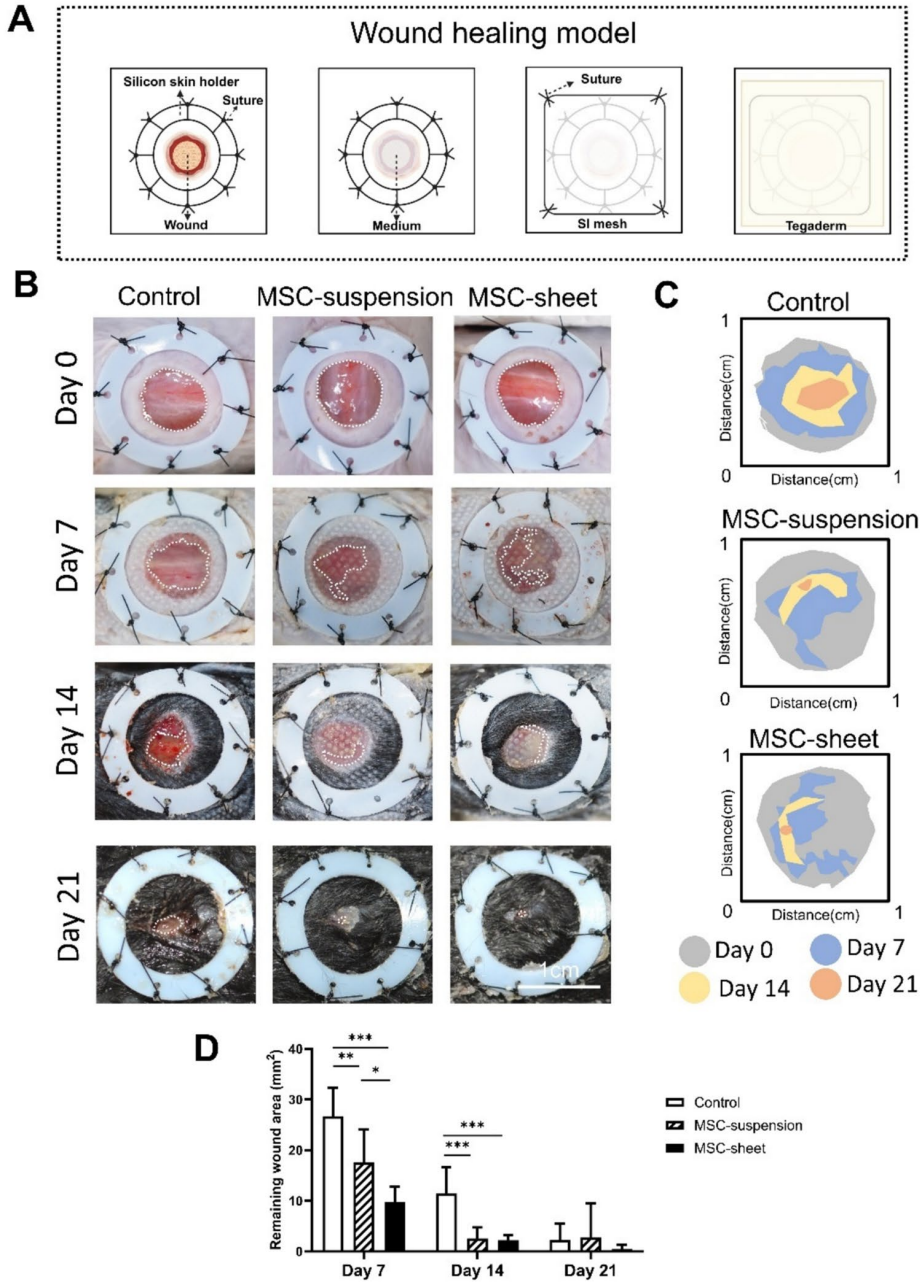
Assessment of the remaining wound area. (**A**) Schematic illustrations of the mouse dorsal skin full-layer defect model. (**B**) Representative photographs showing wound healing progression in different groups on day 0 and after 7, 14, and 21 days of treatment. Scale bars: 1 cm. (C) Overlay maps of wound areas for each group at day 0 (gray), day 7 (blue), day 14 (yellow), and day 21 (orange). (**D**) Quantitative comparison of remaining wound areas on days 7, 14, and 21. On day 7, the MSC-sheet group showed significantly smaller remaining wound areas than the other groups. Moreover, both the MSC-suspension and MSC-sheet groups exhibited faster wound closure than the control group. *p < 0.05, **p < 0.01, ***p < 0.001.

To assess the therapeutic effect, a full-thickness wound-healing model was employed on the dorsal region of mice (Fig. 4A). Wound healing progress was evaluated in mice treated with α-MEM (control), MSC-suspension, and MSC-sheet on days 7, 14, and 21 (Fig. 4B). Wound closure in mice treated with the MSC-suspension and MSC-sheet significantly accelerated on days 7 and 14 compared to that in mice treated with α-MEM, showing no significant difference on day 21 (Fig. 4C). Notably, the wound area in MSC-sheet-treated mice showed a significant reduction on day 7 compared to that in MSC-suspension-treated mice (Fig. 4C), suggesting a more pronounced early-stage healing effect of MSC-sheet treatment.

### Assessment of the neoepithelial length and granulation tissue area

**Fig. 5:**
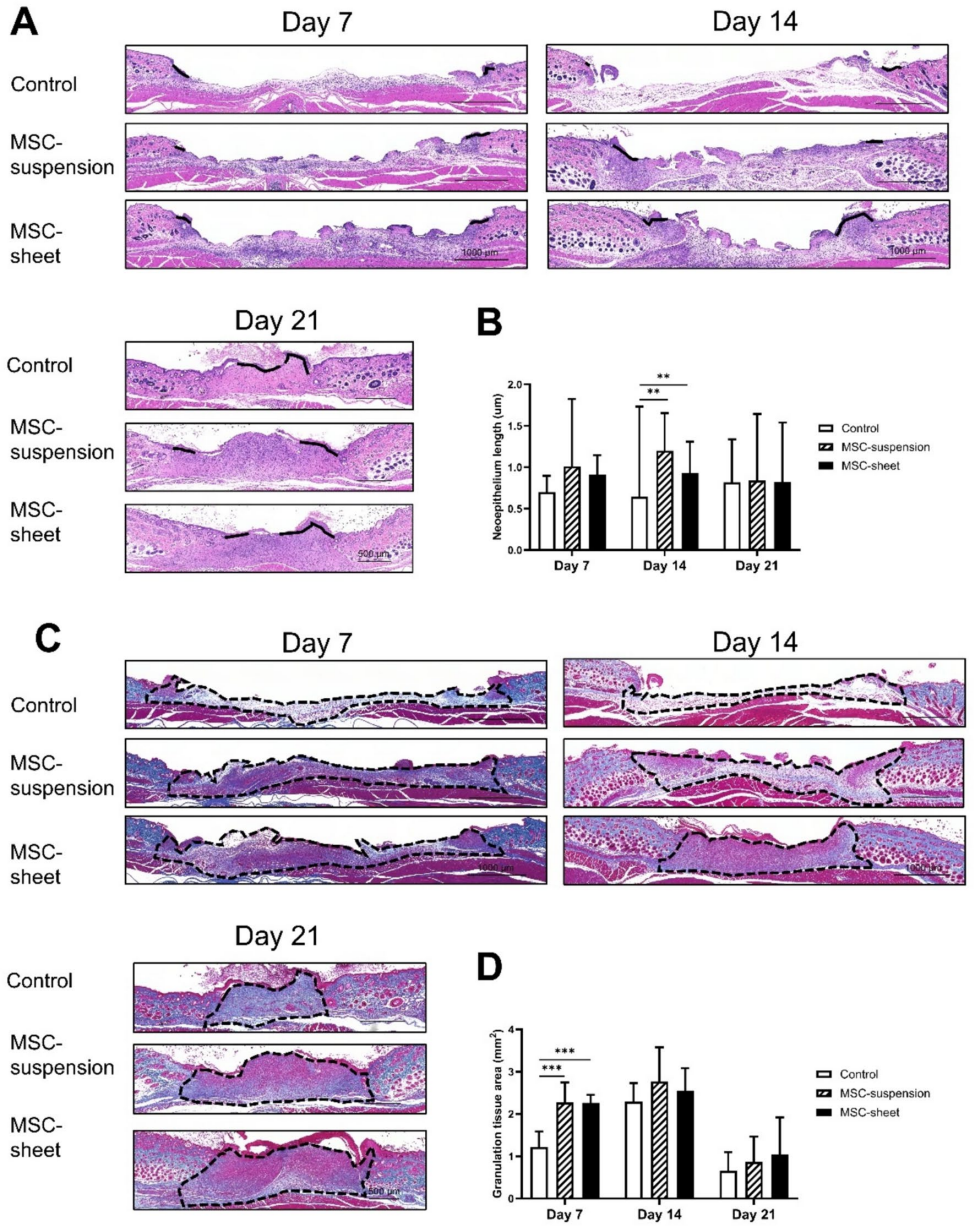
Assessment of the neoepithelial length and newly formed granulation tissue. (**A**) Micrographs of H&E-stained sections on days 7, 14, and 21. The black line indicates the neoepithelium. Scale bar: 1000 μm. (**B**) Quantification of the neoepithelial length. On day 14, both the MSC-sheet and MSC-suspension groups exhibited significantly greater neoepithelial length than the control group. **p < 0.01. (**C**) Micrographs of Azan-stained sections taken on days 7, 14, and 21 Dotted lines outline the newly formed granulation tissue. Scale bar: 1000 μm. (**D**) Quantification of the granulation tissue area. On day 7, both the MSC-sheet and suspension groups showed significantly larger granulation tissue areas than the control group. ***p < 0.001.

Micrographs of H&E-stained sections from the control, MSC-suspension, and MSC-sheet groups on days 7, 14, and 21 are shown in Fig. 5A. On day 14, the neoepithelial length in both the MSC-suspension and MSC-sheet groups was significantly greater than that in the control group (Fig. 5B). The newly formed granulation tissue was evaluated using Azan-stained sections (Fig. 5C). On day 7, both the MSC-suspension and MSC-sheet groups exhibited a significantly larger granulation tissue area than the control group (Fig. 5D). No significant differences in the neoepithelial length and granulation tissue area were identified between the MSC-suspension and MSC-sheet groups throughout the observation period. These findings suggest that the MSC-sheet and MSC-suspension treatments possess comparable capacities for promoting neoepithelial formation and granulation tissue development.

### MSC persistence in wound tissues

**Fig. 6:**
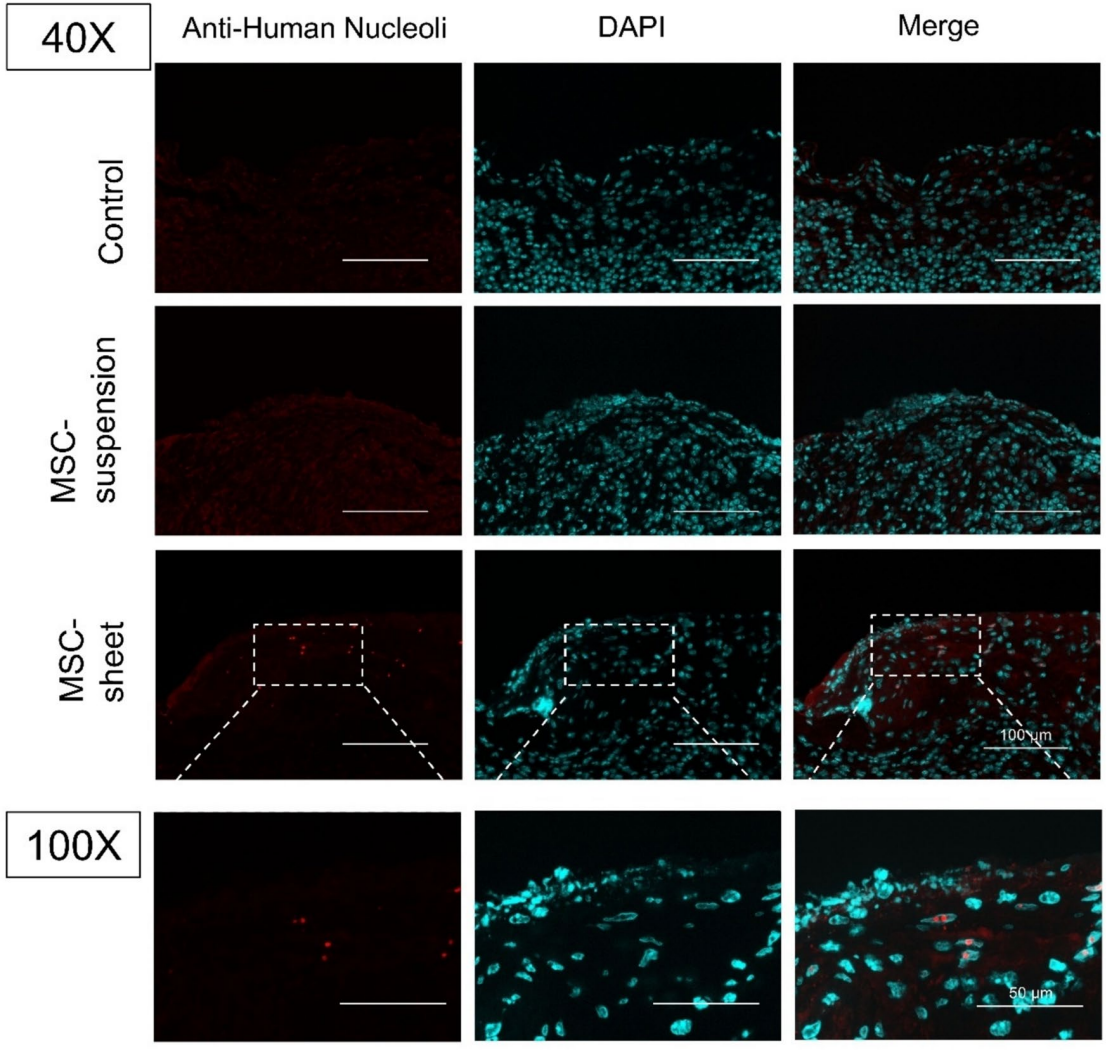
Assessment of MSC retention in the wound tissues. Immunofluorescence micrographs showing human nucleoli (HN)-positive cells (red) and nuclei (DAPI, cyan) on day 7. The merged images demonstrate the presence of HN-positive cells in the MSC-sheet group. Scale bar: 100 μm. High-magnification (100×) images of HN immunofluorescence in the MSC-sheet group. Scale bar: 50 μm.

**Fig. 7:**
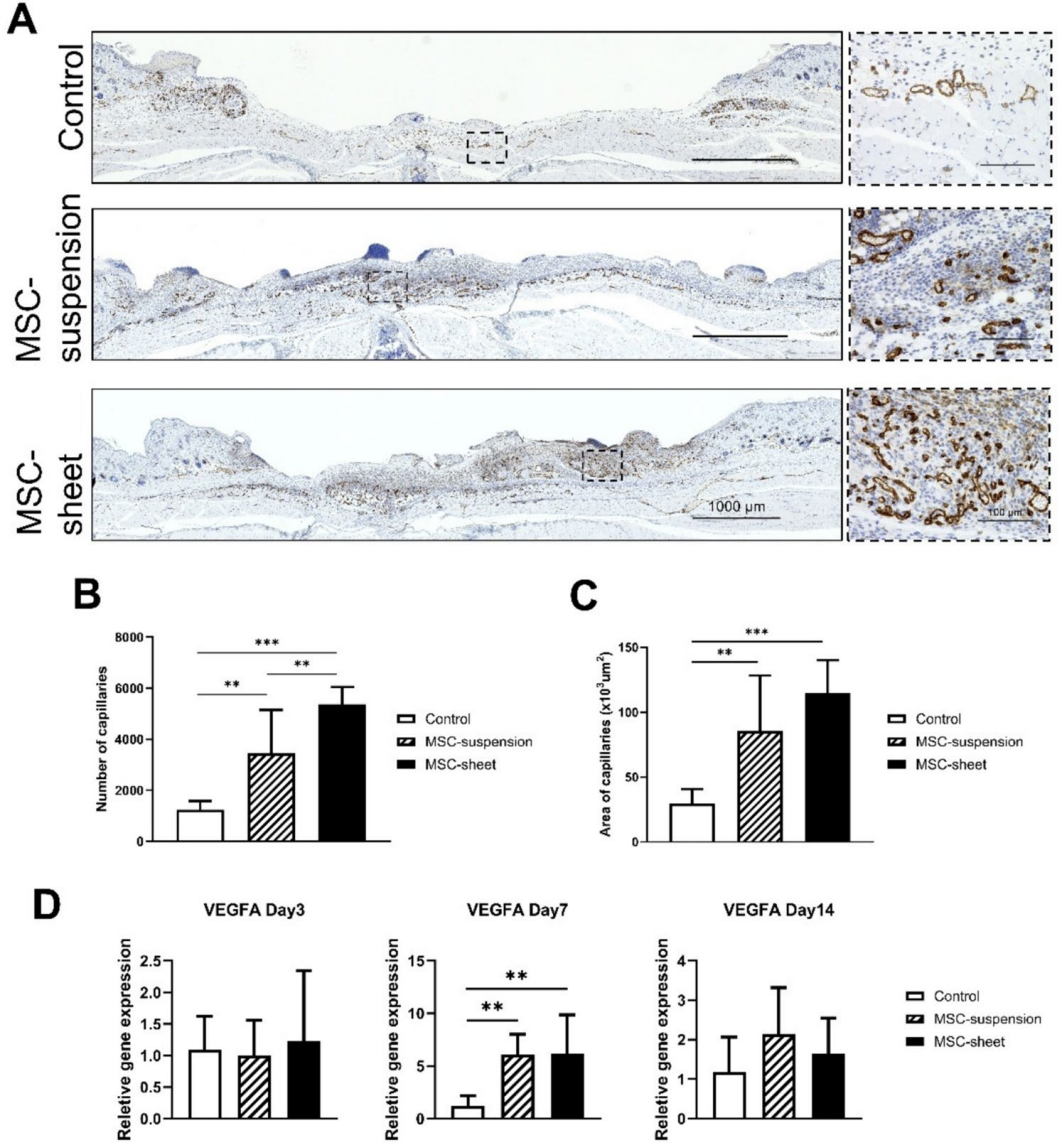
Assessment of newly formed capillaries and VEGFA expression. (**A**) Micrographs of anti-CD31-stained sections showing newly formed capillaries on day 7. Scale bar: 1000 μm. Higher magnification scale bar: 100 μm. (**B**) Quantification of capillary numbers on day 7. Both the MSC-suspension and MSC-sheet groups exhibited significantly higher capillary numbers than the control group, with the MSC-sheet group showing the greatest increase. **p < 0.01, ***p < 0.001. (**C**) Quantification of the area of newly formed capillaries on day 7. The capillary area in both the MSC-suspension and MSC-sheet groups was significantly higher than that in the control group. **p < 0.01, ***p < 0.001. (**D**) Relative VEGFA expression on days 3, 7, and 14. On day 7, VEGFA expression in the MSC-sheet and MSC-suspension groups is significantly higher compared to the control group. **p < 0.01.

To evaluate the *persistence* of transplanted MSCs in the wound area, immunofluorescence staining of HN cells was performed on day 7. Representative micrographs of the control, MSC-suspension, and MSC-sheet groups are shown in Fig. 6. Among the six samples in the MSC-sheet group, five exhibited HN-positive cells (Supplementary Fig. 1), whereas no HN immunofluorescence signals were observed in the MSC-suspension or control groups at the same time points. The high-magnification images (100 ×) in Fig. 7 further demonstrate that the HN marker was localized within the nuclei of the retained cells in the MSC-sheet group. These findings suggest that the MSC-sheet facilitates better cellular persistence in wound tissues compared with the MSC-suspension treatment.

### Assessment of newly formed capillaries

On day 7, anti-CD31 immunostaining was performed to evaluate capillary formation in the wound tissues (Fig. 7A). Quantitative analysis (Fig. 7B, C) revealed that both the MSC-suspension and MSC-sheet groups exhibited significantly increased numbers and areas of newly formed capillaries compared with the control group. Notably, the MSC-sheet group showed the greatest enhancement in capillary number relative to the other groups, indicating that the MSC-sheet confers a superior pro-angiogenic effect at this early stage of wound healing. In addition, the relative VEGFA expression was assessed on days 3, 7, and 14 (Fig. 7D). On day 7, both the MSC-sheet and MSC-suspension groups displayed significantly higher VEGFA levels than the control group, indicating enhanced pro-angiogenic effects of MSC-based treatments.

### Evaluation of macrophage infiltration

**Fig. 8:**
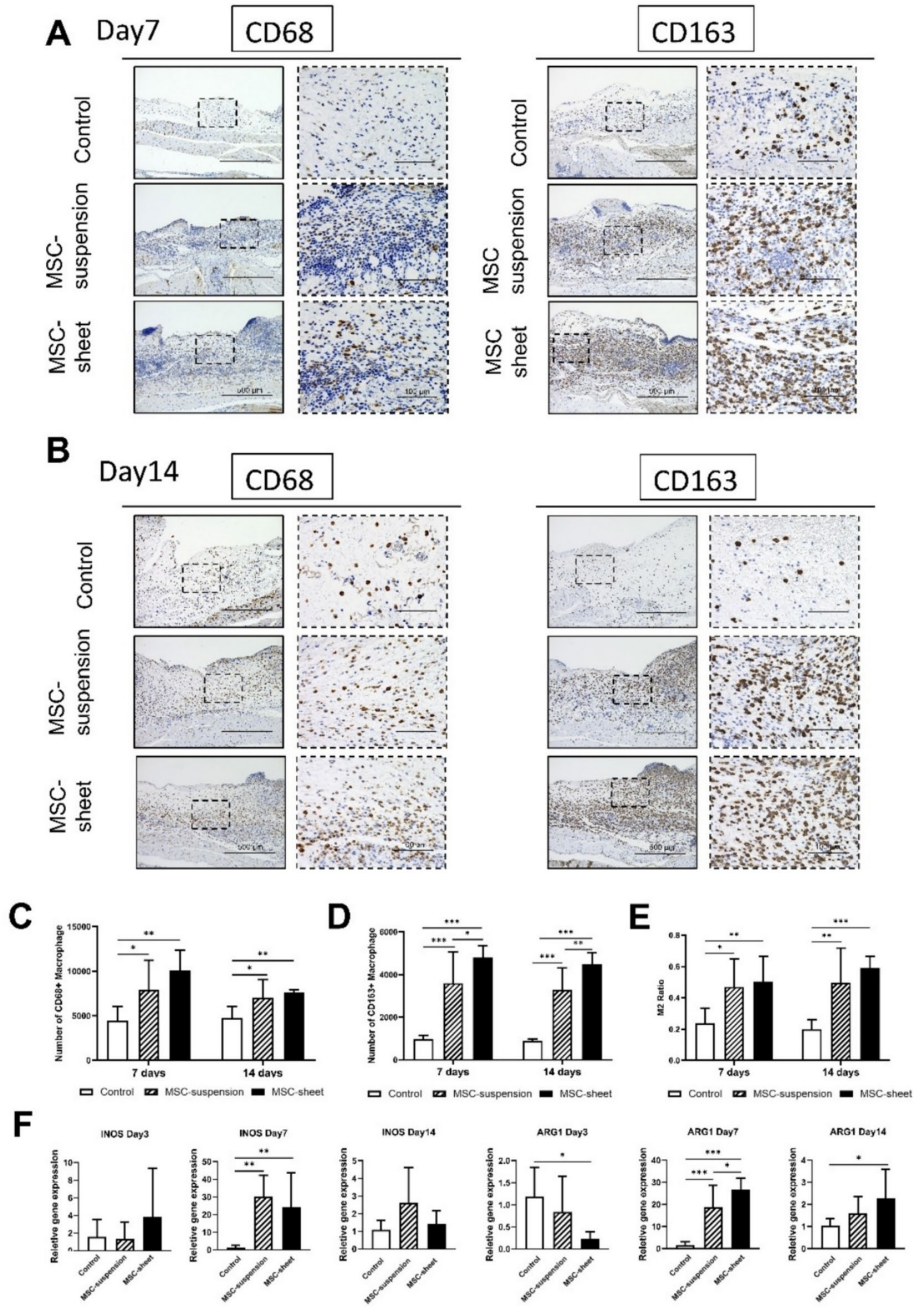
Assessment of macrophage infiltration. (**A**,** B**) Micrographs of wound sections stained with anti‑CD68 (**A**) or anti‑CD163 (**B**) on days 7 and 14. Scale bar: 500 μm; higher-magnification scale bar: 100 μm. (**C**) Quantification of CD68 + macrophages (pan-macrophages) on days 7 and 14. Both the MSC-suspension and MSC-sheet groups exhibited significantly higher numbers of CD68 + macrophages than the control group. ∗*p* < 0.05, ∗ ∗ *p* < 0.01. (**D**) Quantification of CD163 + macrophages (M2 macrophages) on days 7 and 14. The MSC-sheet group exhibited the highest number of CD163 + macrophages, significantly greater than both the control and MSC-suspension groups. *p < 0.05, **p < 0.01, ***p < 0.001. (**E**) Quantification of the M2 macrophage ratio (CD163 + /CD68 +) on days 7 and 14. The MSC-sheet group demonstrated a significantly higher M2 ratio than the control and MSC-suspension groups. *p < 0.05, **p < 01, ***p < 0.001. (**F**) Relative mRNA expression levels of INOS and ARG1 were evaluated by RT-PCR and normalized to GAPDH on days 3, 7, and 14. ARG1 expression in the MSC-sheet group was significantly higher than that in the MSC-suspension group on day 7 and exceeded that of the control group on days 7 and 14. *p < 0.05, **p < 0.01, ***p < 0.01.

Macrophage infiltration was evaluated in wound sections immunostained with anti‑CD68 (pan-macrophages) and anti‑CD163 (M2 macrophages) antibodies on days 7 and 14 (Fig. 8A, B). Quantitative analyses revealed that both the MSC-suspension and MSC-sheet groups had significantly higher numbers of CD68⁺ (Fig. 8C) and CD163⁺ macrophages (Fig. 8D) than the control group at both time points. Moreover, the M2 macrophage ratio (CD163⁺/CD68⁺) was significantly elevated in these two groups compared with the control group (Fig. 8E). Notably, on days 7 and 14, the MSC-sheet group displayed a significantly higher number of CD163⁺ macrophages (Fig. 8D) and a higher M2 ratio (Fig. 8E) than the MSC-suspension group, suggesting that the MSC-sheet more effectively promotes M2 macrophage polarization during wound healing.

### Evaluation of macrophage polarization using RT-PCR

Macrophage polarization was evaluated on days 3, 7, and 14 using RT-PCR, with INOS serving as the M1 marker and ARG1 serving as the M2 marker (Fig. 8F)^31^. On day 3, the MSC-sheet group exhibited significantly lower ARG1 expression compared to the control group, while INOS expression displayed a trend towards increasing levels compared to the control and MSC-suspension groups. This result indicated that the MSC-sheet promoted inflammatory macrophage polarization during the inflammation phase of wound healing. By day 7, both the MSC-suspension and MSC-sheet groups demonstrated higher expression levels of INOS and ARG1 than the control group, indicating increased aggregation of M1 and M2 macrophages. Moreover, ARG1 expression in the MSC-sheet group was significantly higher than that in the MSC-suspension group, suggesting that the MSC-sheet promoted M2 macrophage polarization to a greater extent than the MSC-suspension. On day 14, INOS and ARG1 expressions did not significantly differ between the MSC-suspension and control groups. However, compared with the control group, the MSC-sheet group showed a higher ARG1 expression, suggesting that the MSC-sheet continued to promote M2 macrophage polarization up to day 14 after transplantation.

## Discussion

The present study investigated the therapeutic effects and underlying mechanisms of the self-assembling multilayer MSC-sheet and MSC-suspension in a murine model of wound healing. Both treatments reduced wound area and promoted neoepithelialization, granulation, angiogenesis, and macrophage infiltration. Notably, the MSC-sheet demonstrated enhanced M2 macrophage polarization and superior persistence of transplanted cells, outcomes that directly address key limitations of current MSC therapies.

Maintaining an effective MSC density at the wound site is crucial for therapeutic efficacy. Prior study have shown that transplantation of 1 × 10^6^ MSCs into 6-mm wounds result in superior wound closure and angiogenesis compared to lower cell quantities^32^. However, reported cell densities in MSC-sheet research vary widely, ranging from approximately 4.5 × 10^3^ to 1.1 × 10^6^ cells/cm^2^.^33^. In this study, the MSC-sheet comprised 5.5 × 10^5^ cells for a 6-mm diameter wound, which translates to a high cell density of approximately 1.96 × 10^6^ cells/cm^2^. Furthermore, our multilayer MSC-sheet retained key ECM components collagen I, collagen IV, and elastin, which provide structural support and improve cellular adherence, angiogenesis, and tissue remodeling^26,34,35^. These structural features not only provide mechanical anchorage to the host tissue but also prevent cell washout and reduce anoikis, thereby creating a protective microenvironment that significantly prolongs cell survival compared to suspension delivery^36,37^. Moreover, pre-clinical safety tests have confirmed the safety of the MSC-sheet, with no chromosomal aberrations or colony formation observed in in vitro tumorigenic studies^26^. Together, these attributes highlight the translational potential of the MSC-sheet for clinical wound healing applications.

Macrophages play a central role in wound healing by orchestrating the inflammatory and reparative phases of tissue repair ^38–40^. Our findings demonstrated that both the MSC-sheet and MSC-suspension enhanced M2 macrophage polarization, as evidenced by histological and increased ARG1 expression. M2 macrophages are known to support regeneration by secreting anti-inflammatory and pro-angiogenic factors such as IL-10, VEGF, and TGF-β^35,38–40^. These findings are consistent with prior co-culture studies showing that MSCs induce macrophage polarization via IL-4/IL-13 signaling pathways^38,39^. Although IL-4/IL-13 levels were not directly assessed in this study, our RNA-seq data revealed enrichment of TGF-β and HIF-1 signaling, both closely associated with M2 polarization and wound regeneration. These findings suggest that MSC-sheet exert immunoregulatory effect that enhance reparative macrophage responses, a property particularly relevant to chronic wounds such as diabetic ulcers where impaired polarization contributes to poor healing.

Previous in vitro experiments have reported that MSC-sheet secret higher levels of VEGF and HGF than MSC-suspension^40^. Additionally, macrophages cultured in MSC-sheet-conditioned medium showed higher fluorescence intensity of the M2 macrophage surface marker CD206 and increased CCL18 and IL1RA expressions compared with other cell sheet types^41^. Consistently, our in vivo findings showed that the MSC-sheet significantly enhanced wound closure, vascularization, M2 polarization by day 7 compared with the MSC-suspension. Previous studies have indicated that increased blood supply during the early phase of the repair process, particularly within the first 7 days, can significantly promote the formation of granulation tissue^42^. Therefore, we selected day 7 for CD31 immunostaining to capture the peak of MSC-induced early neovascularization. In our study, we also observed a marked upregulation of VEGFA expression on day 7, further supporting the biological and mechanistic relevance of this time point for evaluating MSC-mediated angiogenesis. The enhanced therapeutic effect of the MSC-sheet may also be attributed to the prolonged presence of transplanted cells at the wound site, which is consistent with the reported cell retention times in the MSC-sheet^43,44^. Additionally, the ECM provided by the MSC-sheet may have facilitated a microenvironment for vascular growth and macrophage infiltration. By day 14, the MSC-sheet continued to exhibit strong ARG1 expression, indicating sustained induction of M2 macrophages.

RNA-seq analysis further supported these findings by identifying 756 DEGs between the MSC-sheet and MSC-suspension. The key upregulated genes in the MSC-sheet, including VEGFA, HGF, and GPNMB, enhanced the angiogenesis and anti-inflammatory responses, which are critical for wound healing. GO and KEGG enrichment analyses revealed the involvement of DEGs in chromosome segregation, cell cycle regulation, tubulin binding, and transmembrane transporter activity. KEGG pathway analysis emphasized the roles of TGF-β and HIF-1 signaling in angiogenesis and macrophage polarization, which are key mechanisms underlying the therapeutic efficacy of the MSC-sheet. PPI network analysis and Cytoscape clustering identified interconnected gene clusters enriched in pathways promoting M2 macrophage polarization and anti-inflammatory responses. These transcriptomic profiles delineate molecular mechanisms that may underlie the superior therapeutic efficacy of the MSC-sheet and suggest how this approach could be translated into clinical benefit by simultaneously promoting vascularization and resolving inflammation.

In contrast to the findings of Yu et al.^24^, who observed that adipose stem cell sheets inhibited the recruitment of macrophages to wound sites in nude mice, our study observed increased numbers of pan and M2 macrophages in MSC-transplanted mice. This difference may be attributable to the application of human-derived MSCs in immunocompetent host mice in our study. Nakao et al.^44^ demonstrated that human-derived MSC-sheets could modulate the local tissue transplant environment via paracrine signaling, exhibiting higher levels of human HGF and TGF-β1 at the transplant site of normal mice. This supports our results of using xenogeneic cell sheets to promote wound healing.

This study represents a preliminary examination of the therapeutic potential of the self-assembling multilayer MSC-sheet in wound healing. From a translational perspective, the MSC-sheet offers several advantages: (1) scaffold-free preparation eliminates concerns of biomaterial degradation; (2) preclinical safety tests confirmed non-tumorigenicity; and (3) the sheet format enables local application to chronic wounds with high cell retention. These characteristics facilitate regulatory approval and clinical scalability. However, several limitations should be acknowledged. First, cell viability was evaluated only on day 7. Future studies will utilize bioluminescent labeling systems to track long-term cell survival and distribution. Second, while the study employed human MSCs in immunocompetent mice, this xenogeneic context may introduce immune confounders. Future work will include immunodeficient models such as NOD-SCID mice to more accurately isolate MSC-specific effects. Third, RT-PCR analyses were performed on whole wound tissue rather than isolated macrophage populations; because the wound samples contain a mixture of diverse cell types and a small proportion of human MSC-derived RNA, the observed upregulation of macrophage markers (such as ARG1 and iNOS) may be subject to a potential dilution effect on murine transcript normalization and could result from either a phenotypic polarization of resident macrophages or simply an increased recruitment of macrophages to the wound site. Third, RT-PCR analyses were performed on whole wound tissues containing both murine host cells and transplanted human MSCs. This xenogeneic context introduces potential confounders: the presence of human RNA may dilute murine transcripts during normalization, and the bulk nature of the analysis precludes distinguishing whether the observed upregulation of macrophage markers (such as Arg1 and iNOS) arises from phenotypic polarization or merely from increased cellular recruitment. Fourth, the transcriptomic findings in this study have not yet been validated at the protein level, future experiments will detect the expression of key targets. Fifth, this study lacks an assessment of the polarization induced by the MSC-sheet in vitro. Therefore, our future research will focus on analyzing the immunomodulatory effects of the MSC-sheet, particularly its role in promoting M2 macrophage polarization and elucidating the underlying signaling pathways. Future work will focus on diabetic wound models and early-phase clinical feasibility studies to establish safety, scalability, and efficacy.

## Conclusion

Both the MSC-sheet and MSC-suspension were effective in accelerating wound healing and promoting M2 macrophage polarization. The MSC-sheet, with its multilayer structure and preserved ECM, outperformed the suspension in enhancing M2 macrophage infiltration and sustaining cell retention. These findings position the self-assembling multilayer MSC-sheet as a clinically relevant, scaffold-free cellular delivery option for chronic wounds. By integrating innovative bioengineering features with demonstrated therapeutic efficacy, the MSC-sheet provides a practical and translatable platform that bridges basic stem cell research with clinical wound care.

## Supplementary Information

Supplementary Information.

## Data Availability

The data and materials supporting the findings of this study are available from the corresponding author upon reasonable request. Processed RNA-seq data supporting this study have been deposited in the EMBL-EBI BioStudies database under accession S-BSST2278.

## References

[1] Hao, Z., Qi, W., Sun, J., Zhou, M. & Guo, N. Review: Research progress of adipose-derived stem cells in the treatment of chronic wounds. *Front. Chem.*10.3389/FCHEM.2023.1094693 (2023). 36860643 10.3389/fchem.2023.1094693 PMC9968763

[2] Krzyszczyk, P., Schloss, R., Palmer, A. & Berthiaume, F. The role of macrophages in acute and chronic wound healing and interventions to promote pro-wound healing phenotypes. *Front Physiol.* **9**(MAY), 419. 10.3389/FPHYS.2018.00419 (2018). 29765329 10.3389/fphys.2018.00419 PMC5938667

[3] Rodrigues, M., Kosaric, N., Bonham, C. A. & Gurtner, G. C. Wound healing: A cellular perspective. *Physiol Rev.* **99**(1), 665–706. 10.1152/PHYSREV.00067.2017 (2019). 30475656 10.1152/physrev.00067.2017 PMC6442927

[4] Dominici, M. et al. Minimal criteria for defining multipotent mesenchymal stromal cells: The international society for cellular therapy position statement. *Cytotherapy* **8**(4), 315–317. 10.1080/14653240600855905 (2006). 16923606 10.1080/14653240600855905

[5] Hassanshahi, A. et al. Adipose-derived stem cells for wound healing. *J. Cell Physiol.* **234**(6), 7903–7914. 10.1002/JCP.27922 (2019). 30515810 10.1002/jcp.27922

[6] Burst, V. R. et al. Poor cell survival limits the beneficial impact of mesenchymal stem cell transplantation on acute kidney injury. *Nephron Exp Nephrol.* **114**(3), e107–e116. 10.1159/000262318 (2010). 19955830 10.1159/000262318

[7] Kim, K., Bou-Ghannam, S., Thorp, H., Grainger, D. W. & Okano, T. Human mesenchymal stem cell sheets in xeno-free media for possible allogenic applications. *Sci. Rep.* **9**(1), 1–12. 10.1038/s41598-019-50430-7 (2019). 31595012 10.1038/s41598-019-50430-7 PMC6783458

[8] Salazar-Noratto, G. E. et al. Understanding and leveraging cell metabolism to enhance mesenchymal stem cell transplantation survival in tissue engineering and regenerative medicine applications. *Stem Cells.* **38**(1), 22–33. 10.1002/STEM.3079 (2020). 31408238 10.1002/stem.3079

[9] Skog, M. et al. The effect of enzymatic digestion on cultured epithelial autografts. *Cell Transpl.* **28**(5), 638. 10.1177/0963689719833305 (2019). 10.1177/0963689719833305 PMC7103596 30983404

[10] Miersch, C., Stange, K. & Röntgen, M. Effects of trypsinization and of a combined trypsin, collagenase, and DNase digestion on liberation and in vitro function of satellite cells isolated from juvenile porcine muscles. *In Vitro Cell Dev. Biol. Anim.* **54**(6), 406–412. 10.1007/S11626-018-0263-5 (2018). 29785535 10.1007/s11626-018-0263-5 PMC5997727

[11] Kolf, C. M., Cho, E. & Tuan, R. S. Mesenchymal stromal cells: Biology of adult mesenchymal stem cells: Regulation of niche, self-renewal and differentiation. *Arth. Res. Ther.* **9**(1), 1–10. 10.1186/AR2116/FIGURES/3 (2007). 10.1186/ar2116 PMC1860068 17316462

[12] Du, S., Zeugolis, D. I. & O’Brien, T. Scaffold-based delivery of mesenchymal stromal cells to diabetic wounds. *Stem Cell Res. Therapy* **13**(1), 1–19. 10.1186/S13287-022-03115-4 (2022). 10.1186/s13287-022-03115-4 PMC9392335 35987712

[13] Lin, Y. C. et al. Evaluation of a multi-layer adipose-derived stem cell sheet in a full-thickness wound healing model. *Acta Biomater.* **9**(2), 5243–5250. 10.1016/J.ACTBIO.2012.09.028 (2013). 23022891 10.1016/j.actbio.2012.09.028

[14] Ardeshirylajimi, A., Delgoshaie, M., Mirzaei, S. & Khojasteh, A. Different porosities of chitosan can influence the osteogenic differentiation potential of stem cells. *J. Cell Biochem.* **119**(1), 625–633. 10.1002/JCB.26223 (2018). 28618050 10.1002/jcb.26223

[15] Imashiro, C. & Shimizu, T. Fundamental technologies and recent advances of cell-sheet-based tissue engineering. *Int. J. Mol. Sci.* **22**(1), 425. 10.3390/IJMS22010425 (2021). 33401626 10.3390/ijms22010425 PMC7795487

[16] Li, Y. et al. Bone marrow macrophage M2 polarization and adipose-derived stem cells osteogenic differentiation synergistically promote rehabilitation of bone damage. *J. Cell Biochem.* **120**(12), 19891–19901. 10.1002/JCB.29297 (2019). 31338874 10.1002/jcb.29297

[17] Chang, D. et al. Engineering of MSCs sheet for the prevention of myocardial ischemia and for left ventricle remodeling. *Stem Cell Res. Ther.* **14**(1), 102. 10.1186/S13287-023-03322-7 (2023). 37098611 10.1186/s13287-023-03322-7 PMC10127056

[18] Sukho, P. et al. Effects of adipose stem cell sheets on colon anastomotic leakage in an experimental model: Proof of principle. *Biomaterials* **140**, 69–78. 10.1016/J.BIOMATERIALS.2017.06.011 (2017). 28628777 10.1016/j.biomaterials.2017.06.011

[19] Kim, K. et al. Allogeneic mesenchymal stem cell sheet therapy: A new frontier in drug delivery systems. *J. Control. Release* **330**, 696–704. 10.1016/J.JCONREL.2020.12.028 (2021). 33347942 10.1016/j.jconrel.2020.12.028

[20] Masuda, S., Shimizu, T., Yamato, M. & Okano, T. Cell sheet engineering for heart tissue repair. *Adv. Drug Deliv. Rev.* **60**(2), 277–285. 10.1016/J.ADDR.2007.08.031 (2008). 18006178 10.1016/j.addr.2007.08.031

[21] Venugopal, B., Shenoy, S. J., Mohan, S., Anil Kumar, P. R. & Kumary, T. V. Bioengineered corneal epithelial cell sheet from mesenchymal stem cells-A functional alternative to limbal stem cells for ocular surface reconstruction. *J. Biomed. Mater. Res. B Appl. Biomater.* **108**(3), 1033–1045. 10.1002/JBM.B.34455 (2020). 31400069 10.1002/jbm.b.34455

[22] Ohki, T. et al. Prevention of esophageal stricture after endoscopic submucosal dissection using tissue-engineered cell sheets. *Gastroenterology*10.1053/J.GASTRO.2012.04.050 (2012). 22561054 10.1053/j.gastro.2012.04.050

[23] Jia, W., He, W., Wang, G., Goldman, J. & Zhao, F. Enhancement of lymphangiogenesis by human mesenchymal stem cell sheet. *Adv. Healthc. Mater.*10.1002/ADHM.202200464 (2022). 35678079 10.1002/adhm.202200464 PMC11932734

[24] Yu, J., Wang, M. Y., Tai, H. C. & Cheng, N. C. Cell sheet composed of adipose-derived stem cells demonstrates enhanced skin wound healing with reduced scar formation. *Acta Biomater.* **77**, 191–200. 10.1016/J.ACTBIO.2018.07.022 (2018). 30017923 10.1016/j.actbio.2018.07.022

[25] Yu, J., Tu, Y. K., Tang, Y. B. & Cheng, N. C. Stemness and transdifferentiation of adipose-derived stem cells using L-ascorbic acid 2-phosphate-induced cell sheet formation. *Biomaterials* **35**(11), 3516–3526. 10.1016/J.BIOMATERIALS.2014.01.015 (2014). 24462360 10.1016/j.biomaterials.2014.01.015

[26] Nagano, N., Hirano, Y., Kimura, M., Morita, H. & Yasukawa, T. Preclinical study of novel human allogeneic adipose tissue-derived mesenchymal stem cell sheets toward a first-in-human clinical trial for myopic chorioretinal atrophy. *Stem Cell Res. Ther.*10.1186/S13287-024-04118-Z (2024). 39716323 10.1186/s13287-024-04118-z PMC11667907

[27] 細胞シート作製方法. Published online May 10, 2011.

[28] Liu, L. & Shi, G. P. CD31: beyond a marker for endothelial cells. *Cardiovasc Res.* **94**(1), 3–5. 10.1093/CVR/CVS108 (2012). 22379038 10.1093/cvr/cvs108

[29] Li, Y. et al. Modified gelatin hydrogel nonwoven fabrics (Genocel) as a skin substitute in murine skin defects. *Regen. Ther.* **23**, 44–51. 10.1016/J.RETH.2023.03.003 (2023). 37090030 10.1016/j.reth.2023.03.003 PMC10119678

[30] Pan, Y. et al. Single-cell RNA sequencing reveals compartmental remodeling of tumor-infiltrating immune cells induced by anti-CD47 targeting in pancreatic cancer. *J. Hematol. Oncol.*10.1186/S13045-019-0822-6 (2019). 31771616 10.1186/s13045-019-0822-6 PMC6880569

[31] Sawaragi, E. et al. Comparisons of the effects of silk elastin and collagen sponges on wound healing in murine models. *Regen Ther.* **24**, 385–397. 10.1016/J.RETH.2023.09.001 (2023). 37719890 10.1016/j.reth.2023.09.001 PMC10502320

[32] O’Loughlin, A. et al. Topical administration of allogeneic mesenchymal stromal cells seeded in a collagen scaffold augments wound healing and increases angiogenesis in the diabetic rabbit ulcer. *Diabetes* **62**(7), 2588–2594. 10.2337/DB12-1822 (2013). 23423568 10.2337/db12-1822 PMC3712062

[33] Kondo, M., Kameishi, S., Grainger, D. W. & Okano, T. Novel therapies using cell sheets engineered from allogeneic mesenchymal stem/stromal cells. *Emerg. Top. Life Sci.* **4**(6), 677–689. 10.1042/ETLS20200151 (2020). 33231260 10.1042/ETLS20200151 PMC7939697

[34] Senk, A. & Djonov, V. Collagen fibers provide guidance cues for capillary regrowth during regenerative angiogenesis in zebrafish. *Sci. Rep.*10.1038/S41598-021-98852-6 (2021). 34593884 10.1038/s41598-021-98852-6 PMC8484481

[35] Gardeazabal, L. & Izeta, A. Elastin and collagen fibres in cutaneous wound healing. *Exp. Dermatol.*10.1111/EXD.15052 (2024). 38483134 10.1111/exd.15052

[36] Miyahara, Y. et al. Monolayered mesenchymal stem cells repair scarred myocardium after myocardial infarction. *Nat. Med.* **12**(4), 459–465. 10.1038/NM1391 (2006). 16582917 10.1038/nm1391

[37] Yang, J. et al. Cell sheet engineering: Recreating tissues without biodegradable scaffolds. *Biomaterials* **26**(33), 6415–6422. 10.1016/j.biomaterials.2005.04.061 (2005). 16011847 10.1016/j.biomaterials.2005.04.061

[38] Wang, J. et al. Transplantation of mesenchymal stem cells attenuates acute liver failure in mice via an interleukin-4-dependent switch to the m2 macrophage anti-inflammatory phenotype. *J. Clin. Transl. Hepatol.* **10**(4), 669–679. 10.14218/JCTH.2021.00127 (2022). 36062289 10.14218/JCTH.2021.00127 PMC9396329

[39] Da Silva, M. L., Bolontrade, M. F., Markoski, M. M., Dallagiovanna, B. & Alaniz, L. Improving the therapeutic ability of mesenchymal stem/stromal cells for the treatment of conditions influenced by immune cells. *Stem Cells Int.*10.1155/2019/6820395 (2019). 31885620 10.1155/2019/6820395 PMC6925793

[40] Bou-Ghannam, S., Kim, K., Kondo, M., Grainger, D. W. & Okano, T. Mesenchymal stem cell sheet centrifuge-assisted layering augments pro-regenerative cytokine production. *Cells* **11**(18), 2840. 10.3390/CELLS11182840/S1 (2022). 36139414 10.3390/cells11182840 PMC9497223

[41] Sukho, P. et al. Human mesenchymal stromal cell sheets induce macrophages predominantly to an anti-inflammatory phenotype. *Stem Cells Dev.* **27**(13), 922–934. 10.1089/SCD.2017.0275 (2018). 29737241 10.1089/scd.2017.0275

[42] Takemoto, S. et al. Preparation of collagen/gelatin sponge scaffold for sustained release of bFGF. *Tissue Eng. Part A.* **14**(10), 1629–1638. 10.1089/TEN.TEA.2007.0215/ASSET/IMAGES/LARGE/FIG-11.JPEG (2008). 18578593 10.1089/ten.tea.2007.0215

[43] Alexandrushkina, N. et al. Cell sheets from adipose tissue MSC induce healing of pressure ulcer and prevent fibrosis via trigger effects on granulation tissue growth and vascularization. *Int. J. Mol. Sci.* **21**(15), 1–21. 10.3390/IJMS21155567 (2020). 10.3390/ijms21155567 PMC7432086 32759725

[44] Nakao, M. et al. Umbilical cord-derived mesenchymal stem cell sheets transplanted subcutaneously enhance cell retention and survival more than dissociated stem cell injections. *Stem Cell Res. Ther.*10.1186/S13287-023-03593-0 (2023). 38072920 10.1186/s13287-023-03593-0 PMC10712142

