# Supplementary material for: Self-assembling multilayer MSC-sheet promotes wound healing increasing M2 macrophage polarization
Source: Sci Rep. 2025 Dec 26;16:3456. doi: 10.1038/s41598-025-33482-w (PMC12834999; doi:10.1038/s41598-025-33482-w)
Supplement: Supplementary file 1 — Supplementary Information. [file 41598_2025_33482_MOESM1_ESM.docx]

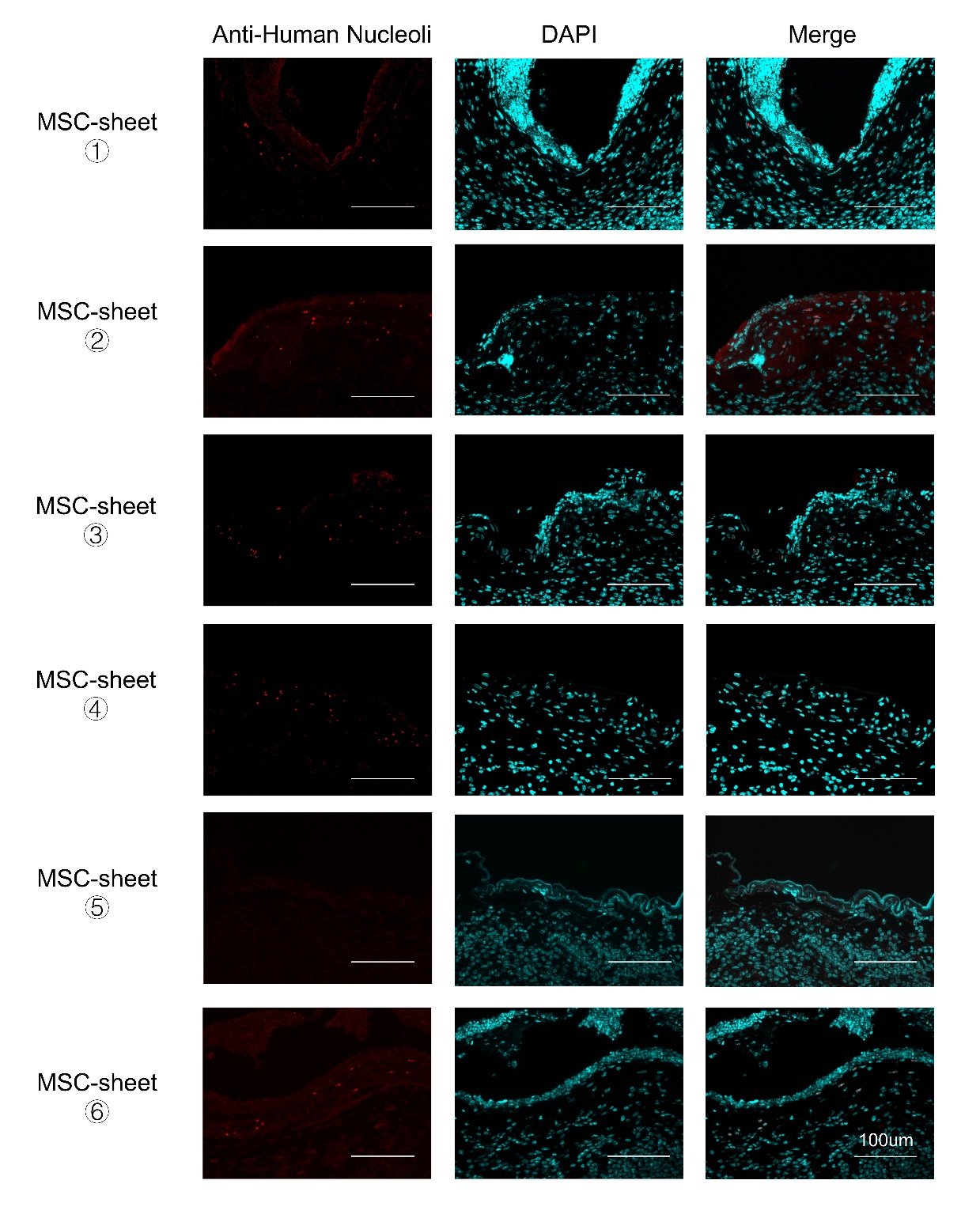


**Supplementary Figure 1**
Animal tissue sections from the MSC-sheet group at one week were stained for human nuclei (HN). Sections numbered 1, 2, 3, 4, and 6 exhibited HN-positive cells, while section number 5 did not show HN-positive cells.
